# Humility is critical in science communication: lessons from the UN’s recent report on child mortality estimates

**DOI:** 10.1186/s41256-025-00444-8

**Published:** 2025-11-08

**Authors:** Daniel D. Reidpath, Brian Wahl, Nina Schwalbe

**Affiliations:** 1 Institute for Global Health and Development, Queen Margaret University, Edinburgh, Scotland, UK; 2 School of Social Sciences, Monash University, Clayton, VIC Australia; 3 Department of Epidemiology of Microbial Diseases, Yale School of Public Health, New Haven, CT USA; 4 Spark Street Advisors, New York, NY USA; 5 Heilbrunn Department of Population and Family Health, Columbia Mailman School of Public Health, New York, NY USA

## Abstract

The United Nations announced a historic milestone in global child mortality in March 2024, with deaths among children less than 5 years falling below 5 million in 2022. While this news is welcome, the headline news is too definitive and masks uncertainties in the results. The UN’s projections rely heavily on modeled estimates based on historical data, with only 5% of the 2022 estimate derived from countries with actual data for that year. The model also fails to account for the potential temporal discontinuity caused by the COVID-19 pandemic and is inconsistent with related data on declining vaccination rates. This critique calls for greater humility in communicating the uncertainties inherent in such projections and emphasizes the need for more robust, empirically grounded estimates to inform global health policy. The UN’s role as a science communicator should be to provide clear, evidence-based insights while acknowledging the limitations of its methodology.

## Background

As the world’s leading authority on human development metrics and global welfare statistics, the United Nations (UN) is a key actor in science communication. UN agencies collect and synthesize data from various sources, including administrative data from member states, academic research, and routine surveys. These agencies then attempt to bridge the gap between the technical details of scientific findings and the practical needs of decision-makers by distilling complex and often messy information into clear, evidence-based narratives that highlight key trends and provide actionable insights. These communications can have a far-reaching impact, influencing policy decisions, resource allocation, and public opinion worldwide.

An announcement in March 2022 by the UN and its agencies demonstrates how their roles as advocates and fundraisers can conflict with their roles as science communicators and evidence-based policymakers [1]. In early 2024, the United Nations Children’s Fund (UNICEF), the World Health Organization (WHO), and the World Bank Group announced a “historic milestone as global child deaths fall below 5 million in 2022” (see [1, 2]). The announcement was based on the top-billed finding from a report by the United Nations Inter-agency Group for Child Mortality Estimation (UN IGME), published concurrently with the media announcement [3]. Unfortunately, while noting that the death toll among children and young people remains unacceptably high, the data tell a story that is much less clear than the headlines would imply. The UN IGME child mortality estimates rely heavily on modeled data from the past, include methodological assumptions that do not fully consider changes over time, and show inconsistencies when compared to other related data. These UN IGME child mortality estimates are also the basis for most cause-specific child mortality estimates. After providing a background on the UN IGME estimates and how they are derived, we briefly discuss these three issues before addressing the implications and providing recommendations.

## UN IGME child mortality approach

UN IGME generated the updated child mortality estimates using nationally representative data from vital registration systems, population censuses, household surveys, and sample registration systems. Household surveys include the UNICEF-supported Multiple Indicator Cluster Surveys (MICS) and the formerly USAID-supported Demographic and Health Surveys (DHS). To arrive at its estimates, the UN IGME employed a Bayesian B-splines bias-adjusted model called the B3 model [3, 4]. This model generates a trend curve of child mortality over time for each country. The model fits all available data points from that country while incorporating information from other countries to fill data voids. Where annual mortality figures are missing within the endpoints of available data, the curve interpolates what the mortality would have been. The model extrapolates mortality estimates to project into the future based on a common reference year to allow for comparability across countries.

## Interpretation challenges

### Over-reliance on modelled estimates

The reliance on extrapolated estimates from the B3 model rather than empirically supported, smoothed data is due to the limited availability of recent data at the period's endpoints. Only 5% of the under-five mortality estimate for 2022 is derived from countries with actual data for that year [5]. When considering countries with data from either 2021 or 2022, the percentage increases to 8%. Given this limited empirical basis, independent validation using alternative data sources—such as real-time health surveillance or administrative records—would be essential for assessing the robustness of these estimates.

As shown in Table 1, the lack of data from specific regions and countries compounds the sparse data problem. Data from Sub-Saharan Africa (SSA), for example, is a critical region because of its disproportionate child mortality, accounting for 58.3% of the total estimated child deaths in 2022. A little more than half of all estimated SSA child deaths in 2022 occurred in just four countries (10.6% from the Democratic Republic of Congo; 6.2% from Ethiopia; 4.6% from Niger; and 29.3% from Nigeria). The most recent empirical data for the Democratic Republic of Congo was from 2010; for Ethiopia, it was from 2016; and for Nigeria, the country with the most significant contribution to child mortality in the world, it was from 2015. Only Niger provided relatively contemporaneous estimates to its mortality estimate—2020.

**Table 1:** Analysis of data^*^ availability by country by year (2018–2022) for the top mortality countries in sub-Saharan Africa (50.5% of deaths) and South Asia (84.9% of deaths)

Country	Data availability by year					Percentage regional deaths 2023
	2018	2019	2020	2021	2022	
*Sub-Saharan Africa*						
Nigeria	No	No	No	No	No	29.1
D.R.C.^†^	No	No	No	No	No	10.9
Ethiopia	No	No	No	No	No	6.1
Niger	Yes	No	Yes	No	No	4.4
*South Asia*						
India	Yes	Yes	Yes	No	No	53.6
Pakistan	No	Yes	No	No	No	31.3
*https://childmortality.org/ ^†^Democratic Republic of Congo						

### Temporal discontinuity

The underlying assumption of any temporal extrapolation is that the model developed on data from one period of time will hold true for future periods. The failure of this crucial assumption underpins analytic techniques that rely on temporal discontinuities to demonstrate exogenous causal effects. the UN IGME did not adjust their estimates post-2020 to account for COVID-19, a major temporal discontinuity, despite the pandemic impacting mortality differently across age groups and countries. Globally, the mortality from COVID was heavily skewed towards older people, and mortality in children under 5 was relatively rare [6]. Nonetheless, there are reasons to anticipate that the pandemic may have indirectly affected child survival due to curtailed health services during shutdowns [7]. Particularly in sub-Saharan Africa, only nine countries provided high-quality child mortality data during 2020–2022, representing just 17% of the region's child deaths. Therefore, the majority of estimates rely on extrapolation without considering potential pandemic-related changes. Although the UN IGME analyzed other sources and reported no widespread excess child mortality, they acknowledged data limitations and urged caution. Nonetheless, their assumption of mortality stability despite data gaps introduces a significant limitation, as it overlooks possible indirect effects of the pandemic on child survival.

### Inconsistency with related data

Data related to factors that are known to affect child mortality have not been considered in the updated UN IGME estimates. It is well-established that childhood vaccinations prevent deaths, and conversely, the failure to vaccinate endangers child lives [8]. First, an unvaccinated child is at greater risk of death due to the vaccine-targeted pathogen directly and indirectly to other pathogens through heterologous effects associated with some live vaccines. Second, any cohort immunity effect is jeopardized when vaccine coverage decreases, placing all children at greater risk of death due to the vaccine-targeted pathogen. Finally, health services for treating unvaccinated children who become ill expend human, financial, and commodity resources unnecessarily, which could contribute to saving other lives. Furthermore, any impact of systemic vaccination failures is likely to be lagged. A child does not die when they miss a vaccination session. Rather, they could die sometime later when or if they contract the otherwise preventable disease, which could be a year or more later. When the pandemic started in 2020, routine immunization services were halted or curtailed in many countries. EPI provides the basic regimen of childhood vaccination to prevent diphtheria, tetanus, pertussis, pneumococcal pneumonia, rotavirus, and measles, among others. Despite evidence of declining vaccination rates in 2020 and 2021, the models do not incorporate these lag effects, nor do they reconcile discrepancies between reported vaccination coverage and actual delivery failures. Additionally, other risk factors like undernutrition [9] and limited access to antenatal care [10], affected by COVID-19, remain unconsidered in the updated estimates, risking an overly optimistic portrayal of child mortality trends.

## Conclusions

The concerns we raise are not directly related to the underlying methodology of the UN IGME report, which has acknowledged the problems we have identified here. Instead, our concern is with how the science has been communicated—with the headline-grabbing announcement of declines in child mortality. If true, the results contradict other UN communications on the impact of missed immunizations during this period. A more measured approach to reporting these findings could have balanced optimism with transparency. Examples of celebratory yet less hyperbolic headlines might include: “UN Report: Global Child Mortality Declines, But Uncertainty Remains” or “UN Child Mortality Estimates Show Progress, but Data Gaps Persist”. A more formal scientific piece would have been of greater utility for researchers and policymakers, particularly if it included sensitivity analyses to examine the impact of lagged data on the estimates. The tendency in global health to over sell success prevents an honest assessment of remaining challenges. We owe it to the children to be honest about mortality rates and the ongoing difficulties in reducing them.UN agencies must embrace transparency in communicating uncertainty, recognizing it as an opportunity to build trust rather than a liability.

## Data Availability

Not applicable.
